# Hyperglycemia alters retinoic acid catabolism in embryos exposed to a maternal diabetic milieu

**DOI:** 10.1371/journal.pone.0287253

**Published:** 2023-08-24

**Authors:** Leo Man Yuen Lee, Yun-chung Leung, Alisa Sau Wun Shum

**Affiliations:** 1 Department of Applied Biology and Chemical Technology, Lo Ka Chung Research Centre for Natural Anti-Cancer Drug Development and State Key Laboratory of Chemical Biology and Drug Discovery, The Hong Kong Polytechnic University, Hong Kong, Hong Kong; 2 School of Biomedical Sciences, The Chinese University of Hong Kong, Hong Kong, Hong Kong; University of Mississippi Medical Center, UNITED STATES

## Abstract

Pregestational diabetes is highly associated with increased risk of birth defects. We previously reported that the expression of *Cyp26a1*, the major catabolizing enzyme for controlling retinoic acid (RA) homeostasis, is significantly down-regulated in embryos of diabetic mice, thereby increasing the embryo’s susceptibility to malformations caused by RA dysregulation. However, the underlying mechanism for the down-regulation of *Cyp26a1* remains unclear. This study aimed to investigate whether elevated maternal blood glucose in the diabetic milieu is a critical factor for the altered *Cyp26a1* expression. Streptozotozin-induced diabetic pregnant mice were treated with phlorizin (PHZ) to reduce blood glucose concentrations via induction of renal glucosuria. Embryonic *Cyp26a1* expression level, RA catabolic activity and susceptibility to various RA-induced abnormalities were examined. To test the dose-dependent effect of glucose on *Cyp26a1* level, early head-fold stage rat embryos of normal pregnancy were cultured *in vitro* with varying concentrations of D-glucose, followed by quantification of *Cyp26a1* transcripts. We found that *Cyp26a1* expression, which was down-regulated in diabetic pregnancy, could be normalized under reduced maternal blood glucose level, concomitant with an increase in RA catabolic activity in embryonic tissues. Such normalization could successfully reduce the susceptibility to different RA-induced malformations including caudal regression, cleft palate and renal malformations. The expression level of *Cyp26a1* in the embryo was inversely correlated with D-glucose concentrations. Diabetic patients suffer from retinopathy, dermopathy, male infertility and increased cancer risk. Coincidentally, RA dysregulation is also associated with these health problems. Our results provided evidence that elevated glucose can down-regulate *Cyp26a1* expression level and disturb RA homeostasis, shedding light on the possibility of affecting the health of diabetic patients via a similar mechanism.

## Introduction

Pregnancies complicated by pregestational diabetes are highly associated with increased risk of birth defects [1]. Despite considerable advances made in diabetic management, the congenital malformation rate associated with diabetic pregnancies remains two times higher than in the general population [2–4]. However, the teratological mechanism of diabetic pregnancy is far from clear. Many studies have shown that the risk of diabetic embryopathy is correlated with maternal blood glucose levels or glycated hemoglobin (HbA1c) levels. Pregnancies of women with blood HbA1c concentrations over 7% at early gestation showed a three- to five-fold increase in malformation rates than normal pregnancies [5]. In contrast, effective glycemic control during preconception and early gestation has generally led to lower frequency of malformations [6, 7]. In animal studies, postimplantation rat embryos cultured in elevated glucose condition developed structural defects that were similar to those found in diabetic pregnancies [8–10], and the stage of organogenesis is the critical period of vulnerability to elevated glucose. Furthermore, β-hydroxybutyrate and somatomedin inhibitors that were found to be increased in diabetic animals could also synergize with glucose to cause maldevelopment [10]. These results suggest that hyperglycemia is a critical factor to potentiate diabetic embryopathy.

Retinoic acid (RA), a bioactive metabolite of vitamin A, is the first morphogen identified [11] and plays a crucial role in embryogenesis. RA concentration must be tightly regulated. It is a teratogen when present in excess during development [12]. Using mouse models, we have demonstrated that embryos derived from streptozotocin-induced diabetic pregnant mice or pregnant mice rendered hyperglycemia via D-glucose injection showed increased susceptibility to caudal regression induced by RA [13]. Both diabetic and hyperglycemic conditions enhance the down-regulation of *Wnt-3a*, a gene that is indispensable for the development of the embryonic caudal region, which suggests that the underlying cellular and molecular changes of the two conditions are similar. On the other hand, a reduction in blood glucose concentrations in diabetic mice could completely abolish this increased susceptibility to RA [14]. These results suggest that elevated glucose in the maternal diabetic milieu is a critical factor responsible for potentiating the teratogenic effect of RA. Ectopic RA in the mouse embryo is removed by the cytochrome P450 family 26 (CYP26) enzymes encoded by *Cyp26a1*, *Cyp26b1* and *Cyp26c1*. All three *Cyp26* genes are expressed in regions of the embryo that require tight RA regulation for normal development, for instance, the neural plate, tailbud, heart and pharyngeal tissues during organogenesis [15, 16]. We have previously found that the expression of RA catabolizing enzyme *Cyp26a1*, which is expressed specifically in the caudal end of the mouse embryo at day 9 of gestation, is significantly down-regulated in embryos of diabetic mice, thereby disrupting RA homeostasis and increasing the embryo’s susceptibility to RA-induced malformations, including cleft palate, neural tube defects and caudal regression [17]. Hence, we propose that hyperglycemia is a key factor that down-regulates *Cyp26a1* expression in diabetic pregnancy. The present study, therefore, investigated the effect of hyperglycemia on *Cyp26a1* expression in embryos and how it disrupted RA catabolism.

## Materials and methods

### Experimental animals and phlorizin treatment

**Fig 1:**
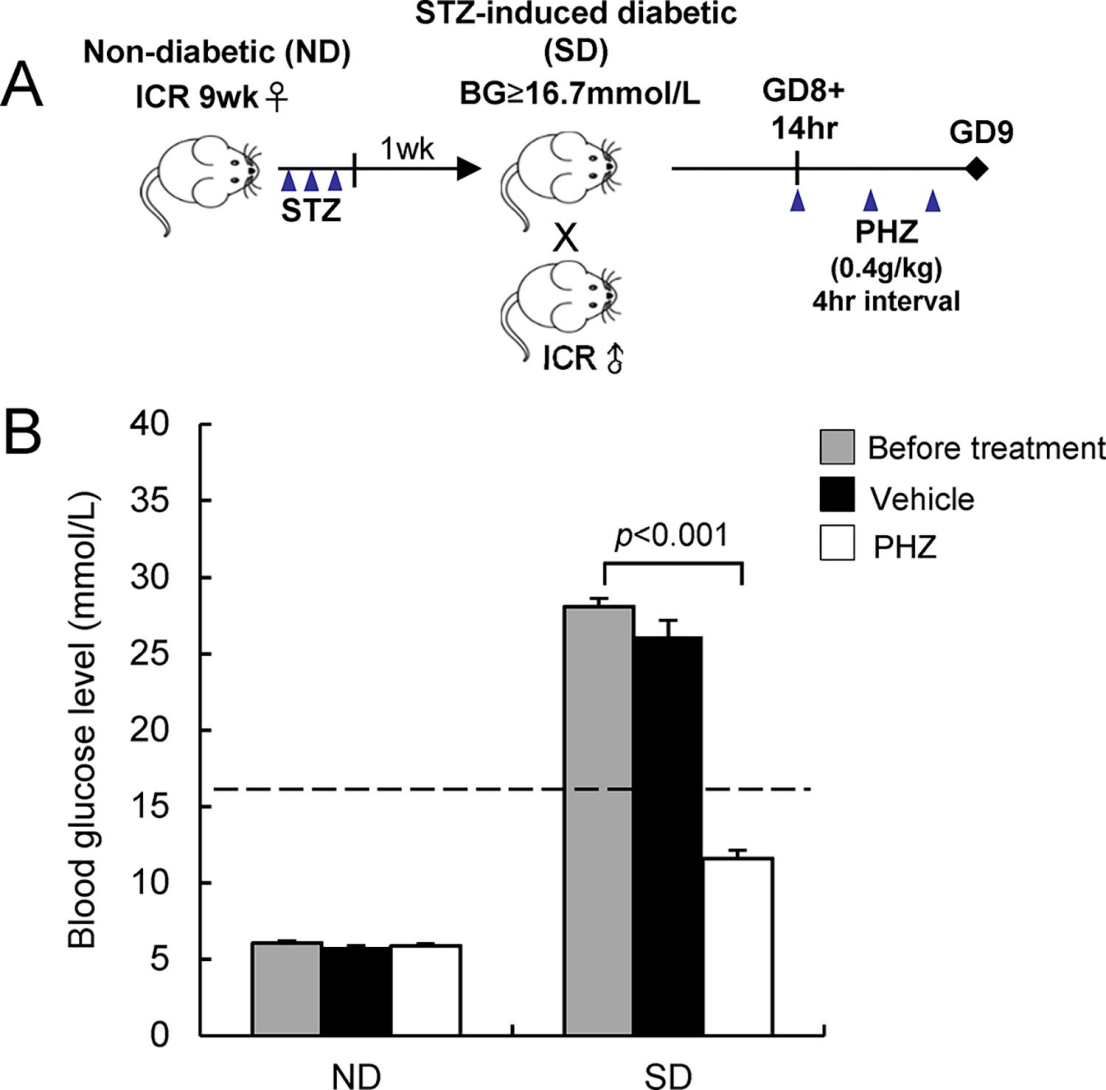
Establishment of a diabetic pregnancy model with transient reduction of blood glucose levels. (A) A schematic diagram showing the experimental design in establishing the model. (B) Blood glucose (BG) levels in pregnant mice before treatment and after treatment with phlorizin (PHZ) or vehicle (CON) for 12 hrs. Animals with blood glucose levels ≥16.7 mmol/L (dotted line) were defined as diabetic. Data are expressed as mean ± SEM of 5–6 mice in each group. Statistical analysis was conducted using paired t-test. GD, gestational day; STZ: streptozotocin.

All animal procedures followed the animal license obtained from The Department of Health, HKSAR (22–516 in DH/HT&A/8/2/1 Pt.35). Experimental procedures were approved by the Animal Experimentation Ethics Committee of The Chinese University of Hong Kong (18/075/GRF-6-U). Type I-like diabetes was induced in 9-week-old female ICR (Institute of Cancer Research) mice with streptozotozin (STZ, *MP Biochemicals*) as previously described [13, 17]. In brief, mice received intraperitoneal injection of STZ (65 mg/kg body weight) for 3 consecutive days. One week after the last dose of STZ, tail vein blood glucose level was measured using a Glucometer Elite (*Bayer*). Mice with blood glucose levels higher than 16.7 mmol/L were regarded as STZ-induced diabetic (SD). SD and age-matched non-diabetic (ND) mice were mated with normal male ICR mice. Phlorizin (PHZ, *Sigma*) was suspended in 40% propylene glycol and administered via intraperitoneal injection at a dose of 0.4 g/kg body weight for 3 injections at 4 hrs intervals starting from hour 14 on gestational day (GD) 8 of pregnancy (GD8+14hr). An equivalent volume of 40% propylene glycol was injected into SD and ND mice to serve as the vehicle control. Blood glucose level was measured prior to dissection (Fig 1A).

### *In vitro* whole embryo culture

Rat embryos, instead of mouse embryos, were employed in this study because the former showed more consistent response to glucose than the latter in *in vitro* culture. Sprague Dawley rats of normal pregnancy were sacrificed by cervical dislocation on GD 9 (equivalent to GD 7.5 of mouse pregnancy). Conceptuses were dissected in a pre-warmed PB1 medium containing 10% fetal bovine serum (*Gibco*). The ectoplacental cone and the yolk sac were left intact. Only embryos at the early head-fold stage were selected for culture *in vitro* in rat serum supplemented with varying concentrations of D-glucose (D-Glu, *BDH*) [2, 3 and 4 mg/mL dissolved in glucose-free DMEM (*Gibco*), which was equivalent to blood glucose levels of 17.0, 22.5 and 28.1 mmol/L respectively] for either 24 or 48 hrs. In the control group, an equivalent volume of DMEM was added. Culture bottles were fitted onto a rotating culture unit inside an incubator (*BTC Engineering*) at 37°C. The culture bottles were continuously aerated with a gas mixture composed of 5% O_2,_ 5% CO_2_ and 90% N_2_ for the first 24 hrs; 20% O_2_, 5% CO_2_ and 75% N_2_ for the next 8 hrs; 40% O_2_, 5% CO_2_ and 55% N_2_ for the remaining 16 hrs. At the end of the culture, embryos were subjected to analysis of the expression pattern of *Cyp26a1* mRNA transcripts by whole-mount *in situ* hybridization [18]. The expression levels of *Cyp26* genes in the embryonic tissues were measured using real-time quantitative RT-PCR.

### *In vitro* RA degrading efficiency

Mouse embryos in different treatment groups were dissected on GD 9 in ice-cold L15 medium (*Gibco*) in the dark under dim yellow light. Only embryos at 19–21 somite-stage were collected. The embryo was divided into 2 parts: the tailbud and the embryonic trunk (defined as the whole body excluding the tailbud) according to the landmark described in S1A Fig. Each lysed sample (consisted of either 4 tailbuds or a single embryonic trunk) was incubated in 50 μL of reaction mixture containing 1.6 mg/mL NADPH (*Sigma*), 0.3 mg/mL DTT (*Sigma*) and 50 nM all-*trans* RA (*Sigma*) [19, 20]. Incubation was carried out in a 5% CO_2_ incubator at 37°C for 2 hrs and protected from light. During incubation, the exogenous RA in the medium was degraded by the RA catabolizing enzyme in the tissue lysate. The amount of RA remaining in the medium was then semi-quantitated using a RA-responsive reporter cell line [21, 22] according to our established protocols [17, 20]. In brief, the RA reporter cells were transfected with a RA response element that drives β-galactosidase expression, and showed a linear response to RA from 10^−6^ to 10^−11^ mol/L. Samples containing RA were added in triplicate to the RA reporter cells grown on a 96-well plate. After 24 hrs of culture, cells were stained with X-gal, and the intensity of the blue-colored product was measured using a microplate spectrophotometer at the wavelength of 600 nm. RA in the sample was quantified using a standard curve constructed with serially diluted RA solutions.

### *In vivo* clearance of RA

On GD 9, ND and SD pregnant mice received an intraperitoneal injection of 25 mg/kg body weight of all-*trans* RA suspended in peanut oil. Embryos were dissected in L15 medium in the dark under dim yellow light at 3 hrs after RA treatment, which had been previously found to be the time point when RA accumulation in the embryo reached the peak level [17]. To compare the RA catabolic activity in different groups, the amount of RA remaining in the tissue of the embryo was determined. Individual tailbuds or embryonic trunks collected from embryos at 20–22 somite-stage were incubated in 300 μL culture medium added with 100 nM CYP26-specific inhibitor (R115866, *Johnson and Johnson Pharmaceutical*) in a 5% CO_2_ incubator at 37°C for 20 hrs to allow the diffusion of RA from tissues into the medium. The concentration of RA in the medium was then determined by the RA-responsive cell line.

### Analysis of different types of RA-induced malformations

SD and ND pregnant mice were treated with PHZ or suspension vehicle as control as previously described. On GD 9, mice were challenged by receiving an intraperitoneal injection of a teratogenic dose of 25 mg/kg body weight of RA. On GD 18, near-term fetuses were examined for the severity of caudal regression by the tail length (TL) to crown-rump length (CRL) ratio. The landmark for measuring TL and CRL in GD 18 fetus was illustrated in S1B Fig. The incidence rates of other types of defects including cleft palate and renal malformations (including hypoplastic, dysplastic, polycystic and horseshoe kidneys, and renal agenesis) were recorded following our previously reported criteria [12]. The occurrence rate was determined by the % of fetuses per litter with the malformation concerned.

### Real-time quantitative RT-PCR

Total RNA was extracted from embryonic tissue using the Total RNA Extraction Kit (*Favorgen*) and reverse transcribed into first-strand cDNA using the High Capacity cDNA Reverse Transcription Kit (*Applied Biosystems*) according to the manufacturer’s protocol. The cDNA synthesized was subjected to real-time quantitative polymerase chain reaction (PCR) using Power SYBR Green Master Mix (*Applied Biosystems*) and ABI 7900 Fast Real-Time PCR system (*Applied Biosystems*) following the manufacturer’s protocol. The PCR primers were designed according to GeneBank sequences of mouse or rat *Cyp26a1*, *Cyp26b1*, *Cyp26c1*, *Tbx1* and *β-actin* and summarized in Table 1.

**Table 1:** Summary of the primer sequence for real-time quantitative RT-PCR.

	Gene	Forward	Reverse
Mouse	*Cyp26a1*	CAG TGC TAC CTG CTC GTG AT	AGA GAA GAG ATT GCG GGT CA
	*Cyp26b1*	TTC AGT GAG GCA AGA AGA CA	CTG GA GGA GGT GCT AAG TA
	*Cyp26c1*	GGG ACC AGT TGT ATG AGC AC	AGC CAA CTC CTT CAG CTC TT
	*Tbx1*	CAG CAG CCA ACG TGT ACT C	TCG GTC GTC TAC ACT GCA AT
	*β-actin*	TGT TAC CAA CTG GGA CGA CA	GGG GTG TTG AAG GTC TCA AA
Rat	*Cyp26a1*	GTG CCA GTG ATT GCT GAA GA	AGA GAA GAG ATT GCG GGT CA
	*Cyp26b1*	CAC ATC CTT GAT CAT GCA AC	AGC CTC ATG ACC TCC TTG AT
	*Cyp26c1*	ATC CCT TAT CCT GCT GCT TC	AGC ACC TCC TTC ACT ACG GC
	*β-actin*	GGA AAT CGT GCG TGA CAT TA	AGG AAG GAA GGC TGG AAG AG

### Statistical analysis

Data on blood glucose concentrations were analyzed by paired t-test. Embryonic *Cyp26* expression levels, RA catabolic activity and susceptibility to various RA-induced abnormalities were analyzed by two-way ANOVA, followed by the Bonferroni test. The correlation between glucose concentration and *Cyp26a1* expression was analyzed by Pearson’s correlation test. All statistical analyses were conducted using SPSS software (*SPSS*, Chicago, Ill., USA), with statistical significance set at a *p*-value of less than 0.05.

## Results

### Establishment of diabetic pregnancy mouse model with reduced blood glucose level

Mice that had undergone streptozotocin treatment showed high blood glucose levels, reaching 28 mmol/L. The blood glucose level of streptozotocin-induced diabetic (SD) mice was significantly (*p* < 0.001) lowered by more than half to a non-diabetic (ND) level (<16.7 mmol/L) after injection of 3 doses of PHZ. Injection of the vehicle (CON) alone did not affect the maternal blood glucose level. ND mice did not show any responses to PHZ treatment and maintained a normal blood glucose level (Fig 1B).

### Normalization of *Cyp26a1* expression in embryos upon reduction of blood glucose levels

**Fig 2:**
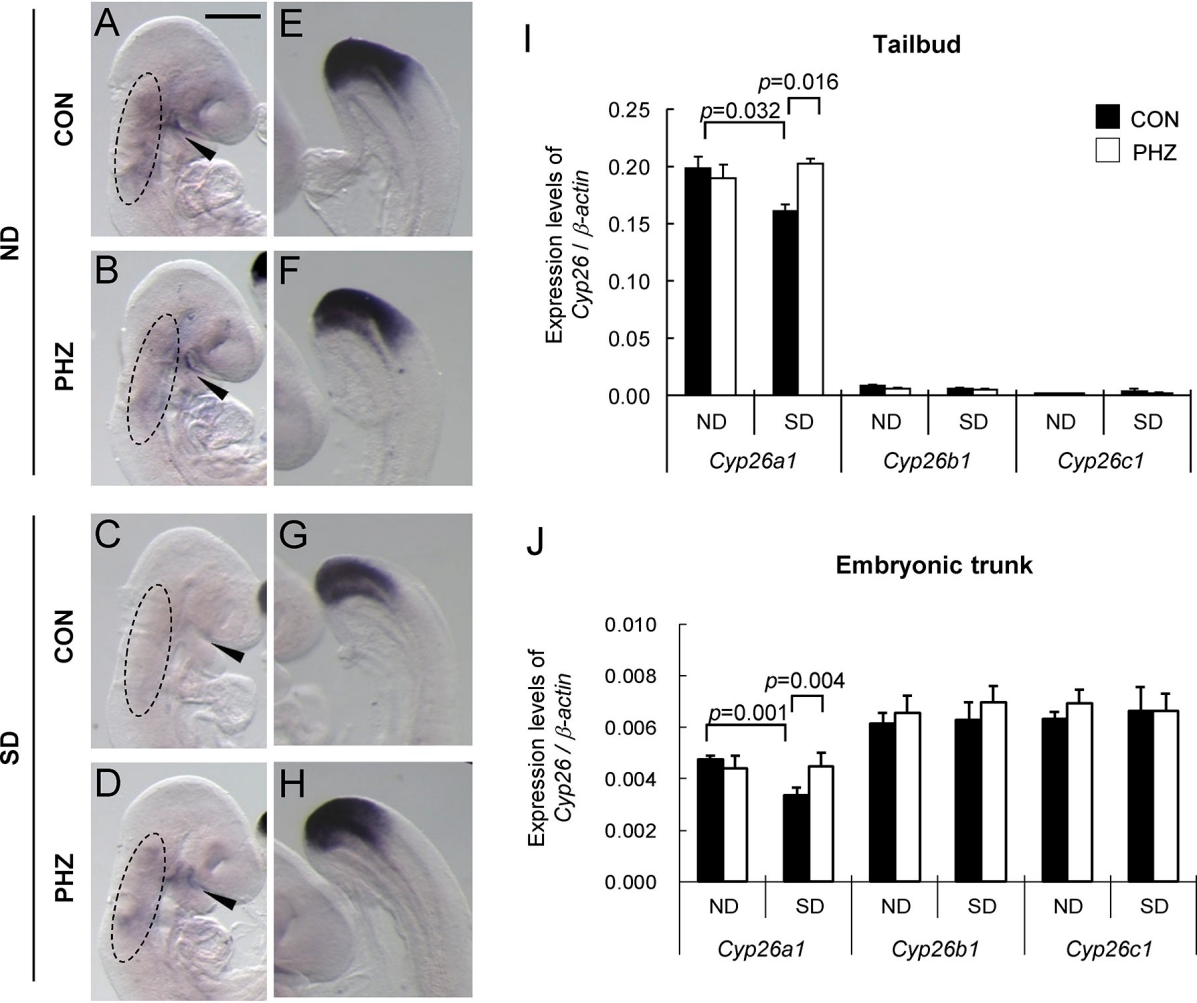
Reduction of maternal blood glucose level by PHZ treatment up-regulates *Cyp26a1* expression but not *Cyp26b1* and *Cyp26c1* expressions. (A-H) Representative embryos showing the expression of *Cyp26a1*, detected by whole-mount *in situ* hybridization, in GD 9 mouse embryos after treatment of PHZ or an equivalent volume of vehicle as control (CON). (A-D) In the anterior region of embryos, *Cyp26a1* was expressed in the facial and cervical mesenchyme (circled) and along the maxillary-mandibular cleft (arrowhead). (E-H) In the posterior region of embryos, *Cyp26a1* was highly expressed in the tailbud region. About 15–20 embryos from 3 litters were examined in each group. Scale bar representing 0.5 mm in A-D and 0.1 mm in E-H. (I-J) The mRNA expression levels of *Cyp26a1*, *Cyp26b1* and *Cyp26c1* relative to *β-actin* in tailbud region of embryos (I) and embryonic trunk (J). The tailbud region was defined as the region from the caudal end to the presomitic region at a level one-somite length caudal to the last somite. Data are expressed as mean ± SEM of 5 litters in each group (embryonic tissues from the same litter were pooled as 1 sample). Statistical analysis was conducted using two-way ANOVA followed by Bonferroni test.

We then examined the embryonic *Cyp26a1* expression using *in situ* hybridization and quantitative RT-PCR (qRT-PCR). Results of qRT-PCR showed that *Cyp26a1* was the most dominant RA degrading enzyme expressed in the tailbud region of embryos while *Cyp26b1* and *Cyp26c1* were hardly found in that region (Fig 2I), which suggested that the RA level in the tailbud region was controlled by *Cyp26a1*. For animals treated with vehicle alone, *Cyp26a1* expression in the tailbud region of embryos in the SD group (Fig 2G and 2I) was significantly (*p* = 0.032) down-regulated when compared with that of the ND group (Fig 2E). With the reduction of maternal blood glucose levels by PHZ injection, the expression of *Cyp26a1* was restored to a level similar to the ND group (Fig 2H). In line with a stable maternal blood glucose level, the expression of *Cyp26a1* did not show any variations in the ND group treated with PHZ (Fig 2F). *Cyp26b1* and *Cyp26c1* did not show any significant changes in all groups (Fig 2I), which supported that the normalization of *Cyp26a1* expression was highly specific.

In the embryonic trunk (whole embryo excluding the tailbud), *Cyp26a1*, *Cyp26b1* and *Cyp26c1* were expressed at comparable levels (Fig 2J). Similar to the tailbud region, a significant (*p* = 0.001) down-regulation of *Cyp26a1* was found in the anterior region of embryos of the SD group that were treated with vehicle (Fig 2C and 2J). Embryos of the SD group showed normalization of *Cyp26a1* expression upon reduction of maternal blood glucose levels by PHZ (Fig 2D). For *Cyp26b1* and *Cyp26c1*, there were no significant differences in expression levels between embryos of ND and SD groups, further supporting that normalization of *Cyp26a1* expression was gene-specific.

### Normalization of RA degrading efficiency in embryos following reduction of maternal blood glucose levels

**Fig 3:**
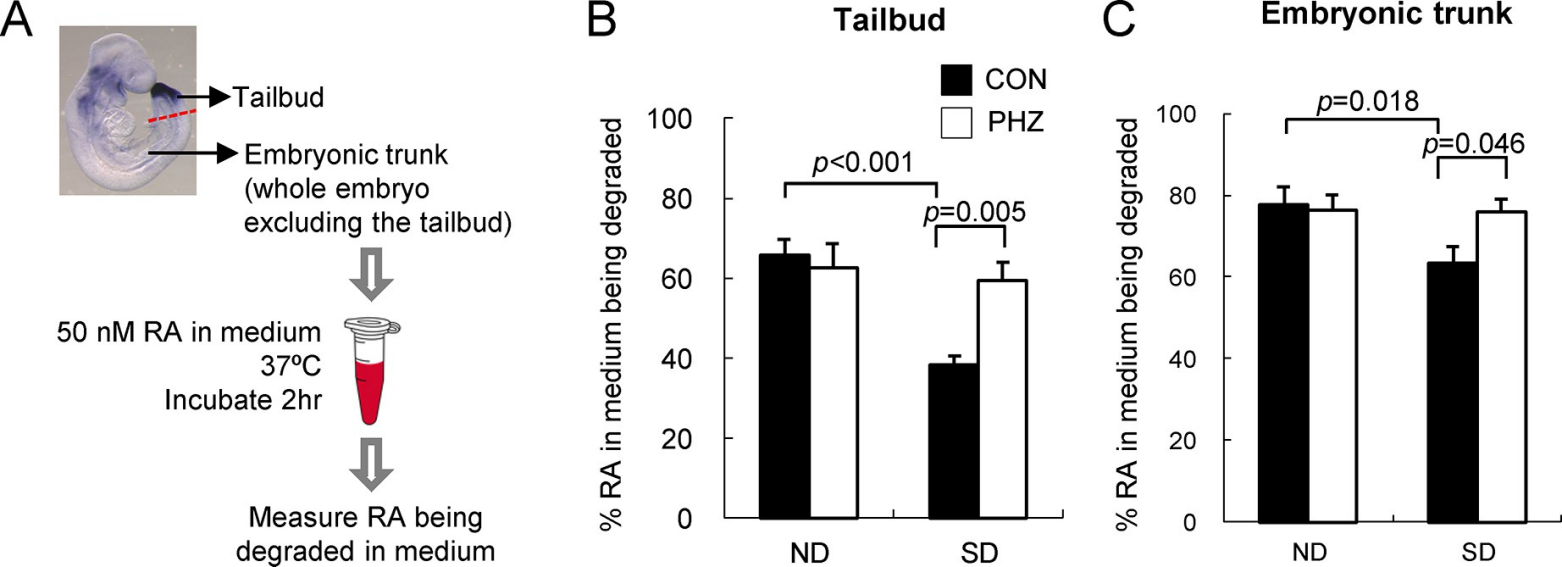
Normalization of RA degrading efficiency in different embryonic tissues upon reduction of maternal blood glucose levels. (A) A schematic diagram showing the experimental design of measurement of *in vitro* RA degrading efficiency. (B-C) RA degrading efficiency in the tailbud region of embryos (B) and embryonic trunk (C). Tissues were incubated in 50 nM RA with 1.6 mg/mL NADPH and 0.3 mg/mL DTT for 2 hrs. Data are expressed as mean ± SEM of 5–6 reactions for the tailbud region and 18 reactions for the embryonic trunk. Statistical analysis was conducted using two-way ANOVA followed by Bonferroni test.

To examine whether the altered *Cyp26a1* expressions have any functional significance in embryos, we measured the RA degrading efficiency *in vitro* (Fig 3A). As previously shown, *Cyp26a1* is the predominant RA degrading enzyme in the tailbud region (Fig 2I), therefore, the RA degrading activity detected was mainly contributed by *Cyp26a1*. The *in vitro* RA degrading efficiency in the tailbud region, represented by the percentage of RA added in the medium being degraded, was found to be significantly (*p* < 0.001) reduced in the SD (CON) group when compared with that of the ND (CON) group (Fig 3B). Upon lowering of blood glucose levels in SD mice with PHZ, the RA degrading efficiency in the tailbud region of their embryos increased significantly (*p* = 0.005) compared with the SD (CON) group.

For the embryonic trunk excluding the tailbud, *Cyp26a1*, *Cyp26b1* and *Cyp26c1* were co-expressed in the anterior region of the embryo (Fig 2J). A trend similar to the finding of the tailbud region was observed. The SD (CON) group showed a significantly (*p* = 0.018) lower RA degrading efficiency than the ND (CON) group. However, RA degrading efficiency of the SD group was corrected to a level similar to the ND (CON) group after treatment of PHZ (Fig 3C). These results were complementary with the finding of *Cyp26a1* expression, i.e. once the *Cyp26a1* expression level was normalized, there was a corresponding normalization of the *in vitro* RA degrading activity.

### Reduction of susceptibility to various RA-induced malformations

**Fig 4:**
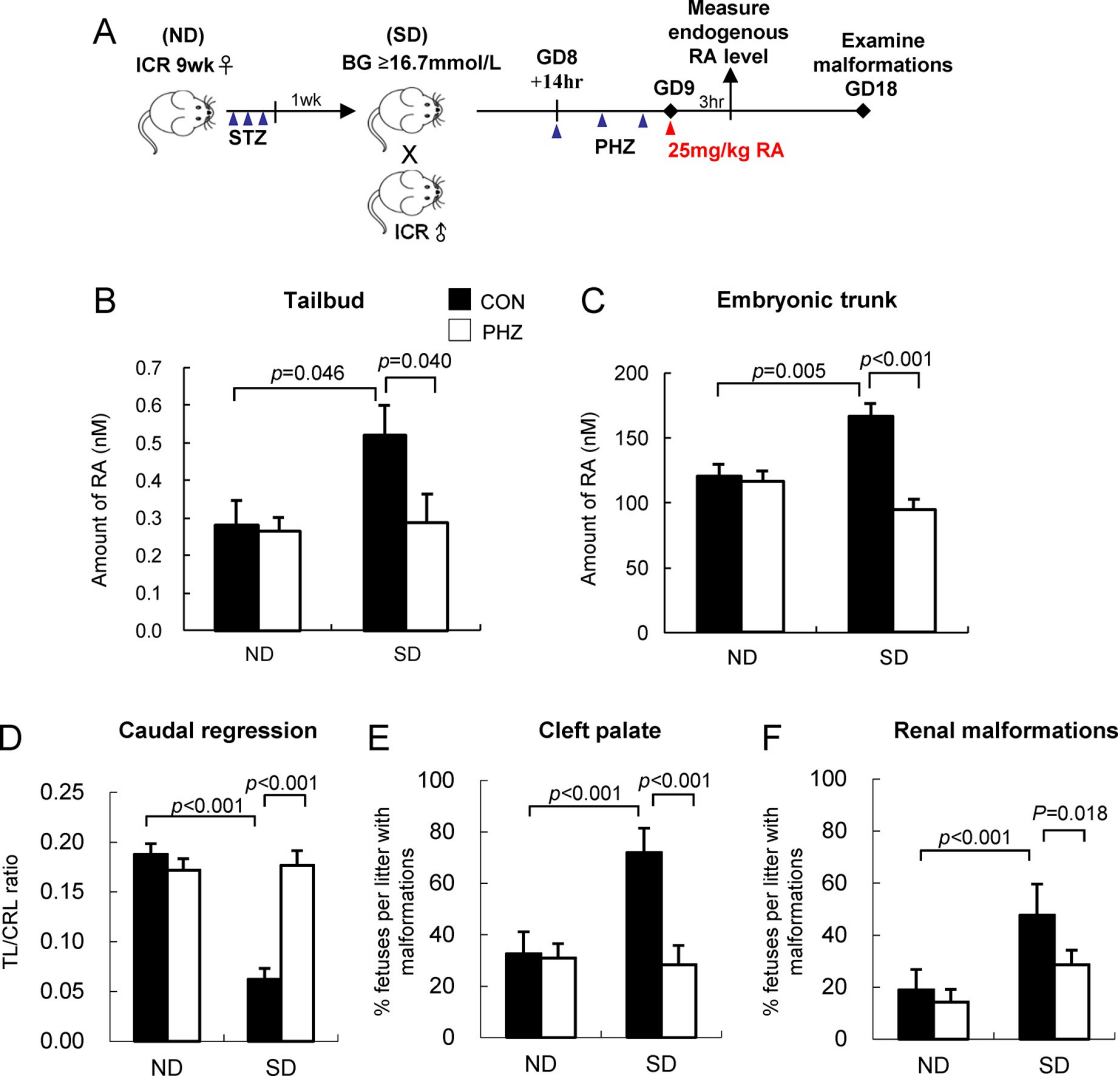
Reduction of maternal blood glucose reduces RA levels in tissues and abolishes the increased embryonic susceptibility to various RA-induced malformations in diabetic pregnancy. (A) A schematic diagram showing the experimental design. (B-C) Amount of RA in the tailbud region of embryos (B) and embryonic trunk (C) at 3 hrs after injection of 25 mg/kg dose of RA. Data are expressed as mean ± SEM of 11–12 individual tailbud/embryonic trunk examined for each group. (D) The severity of caudal regression is represented by TL/CRL ratio. (E-F) Percentage of near-term fetuses per litter with cleft palate (E) and renal malformations (F). Data are expressed as mean ± SEM of 7–8 litters for each group. Statistical analysis was conducted using two-way ANOVA followed by Bonferroni test.

We further examined the RA catabolizing activity in embryos *in vivo* by challenging the embryos with 25 mg/kg RA on GD 9 (Fig 4A). At 3 hrs after RA treatment, embryos in the SD group treated with vehicle had a significantly higher level of RA than the ND group in both the tailbud (Fig 4B) and the embryonic trunk (Fig 4C), which suggested that exogenous RA had accumulated in these tissues. With the upregulation of *Cyp26a1* expression by reduction of maternal blood glucose using PHZ, the amount of RA present in the embryonic tissues of the SD group was significantly (*p* = 0.040 for tailbud; *p* < 0.001 for embryonic trunk) reduced. This result was in line with the *in vitro* RA degrading activity (Fig 3B and 3C), which supported that the increased *Cyp26a1* expression and RA degrading efficiency by reducing maternal blood glucose level could help to defend against the aberrant buildup of RA in embryos.

To further investigate whether normalization of *Cyp26a1* expression by transient reduction of maternal blood glucose levels with PHZ could indeed reduce embryos’ susceptibility to RA teratogenesis, pregnant mice were challenged with a teratogenic dose of 25 mg/kg RA on GD 9 to induce different types of malformations, including cleft palate, kidney malformations and caudal regression. These malformations are shown to exhibit increased incidences in diabetic pregnancy [5, 17]. Near-term fetuses of the SD (CON) group had significantly reduced TL/CRL ratio (*p* < 0.001; Fig 4D), which meant more severe caudal regression, and showed higher incidence rates of cleft palate (*p* < 0.001; Fig 4E) and renal malformations (*p* < 0.001; Fig 4F) in comparison with fetuses of the ND (CON) group. In contrast, lowering of maternal blood glucose levels by PHZ treatment could rescue the adverse effect of ectopic RA in the SD group (Fig 4B and 4C), and ameliorate the severity of caudal regression, as shown by the increased TL/CRL ratio (*p* < 0.001), and reduced incidence rates of cleft palate (*p* < 0.001) and renal malformations (*p* = 0.018) in near-term fetuses. Taken together, these results demonstrated that the susceptibility of *Cyp26a1*-normalized embryos of the SD group to various types of RA-induced malformations was significantly reduced such that increased embryonic susceptibility to RA teratogenicity caused by diabetic pregnancy was completely abolished.

### Dose-dependent down-regulation of *Cyp26a1* by glucose

**Fig 5:**
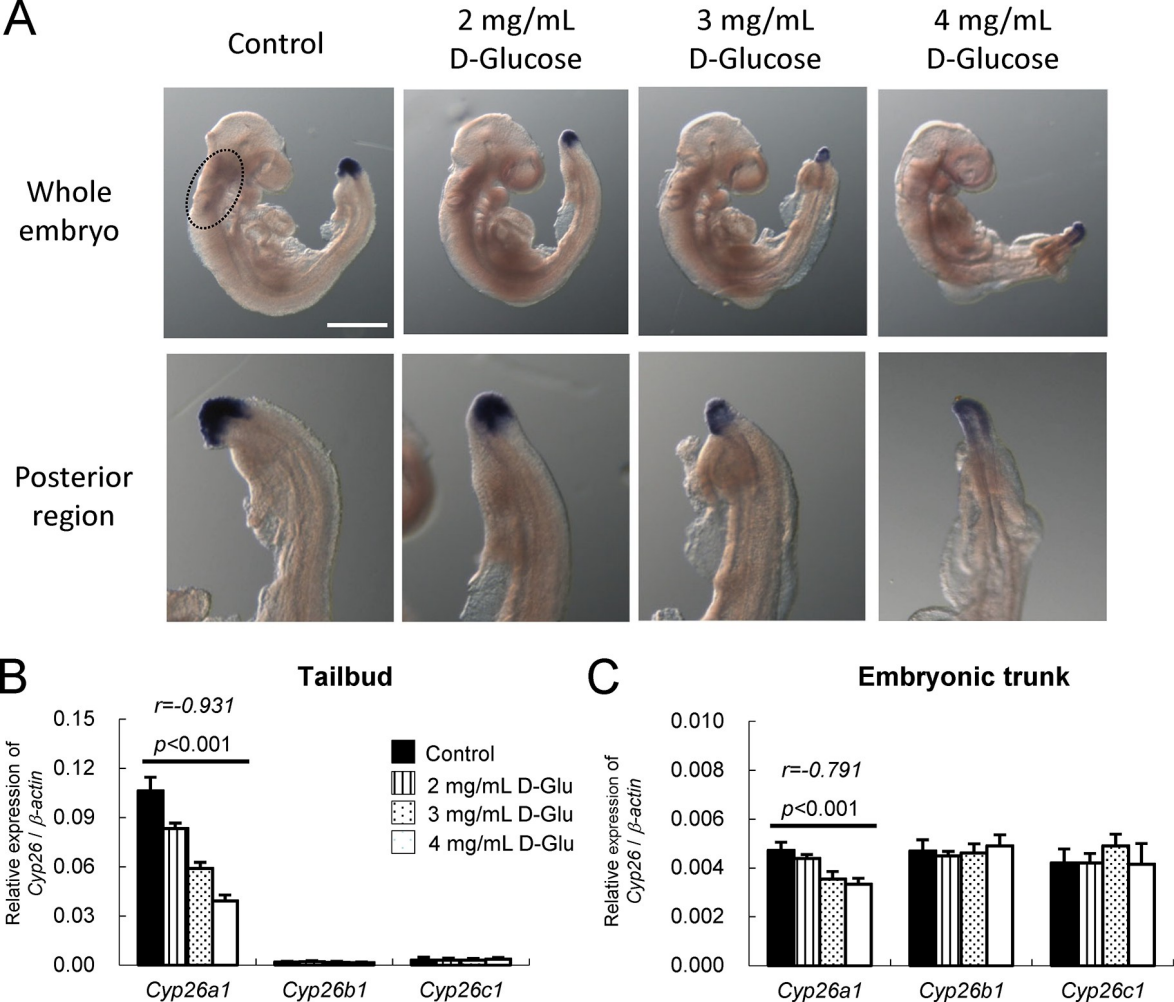
Whole embryo culture of GD 9 rat embryos in varying concentrations of D-glucose for 48 hrs demonstrates a dose-dependent suppression of *Cyp26a1* and caudal regression. (A) Representative embryos showing the expression of *Cyp26a1* in the anterior and posterior regions of embryos detected by whole-mount *in situ* hybridization. Dose-dependent down-regulation of *Cyp26a1* in rat embryos cultured in serum supplemented with different concentrations (2, 3 and 4 mg/mL) of D-glucose (D-Glu) or DMEM (Control) for 48 hrs from GD 9 (equivalent to GD 7.5 of mouse embryo). Around 20–25 embryos from 3–4 litters in each group were examined. Cranial and cervical mesenchyme were circled. Scale bar representing 0.5 mm for whole embryo and 0.25 mm for posterior region. (B-C) The mRNA expression levels of *Cyp26a1*, *Cyp26b1* and *Cyp26c1* relative to *β-actin* in tailbud region of embryos (B) and embryonic trunk (C). Data are expressed as mean ± SEM with 7 samples for each group. Embryonic tissues from 3 embryos were pooled as one sample. Statistical analysis was conducted using Pearson’s correlation test.

To further determine if glucose could regulate *Cyp26a1* expression in a dose-dependent manner, whole embryo culture, which allowed accurate control of glucose concentrations in the culture medium, was employed. Rat embryos were cultured in rat serum supplemented with varying concentrations of D-glucose (2, 3, and 4 mg/mL) or an equivalent volume of DMEM as vehicle control (Control). After *in vitro* culture for 24 hrs from the head-fold stage, embryos without D-glucose supplementation (Control) developed normally into the early somite stage. Results of an *in situ* hybridization study showed that *Cyp26a1* was normally expressed in the posterior neural plate and the tailbud mesoderm in the caudal region (S2A Fig). There was a weak expression in the anterior region near the optic primordium (S2A Fig, red arrowhead). For embryos cultured in serum supplemented with 2 mg/mL D-glucose, there was a significant reduction of *Cyp26a1* expressions in both the tailbud region and the anterior region (Fig 2A–2C). As the concentration of D-glucose increased to 3 mg/mL and 4 mg/mL, *Cyp26a1* expression in the posterior neural plate appeared to have diminished, and *Cyp26a1* expression was shifted to a more ventral part at the caudal limit. After being cultured for 48 hrs, *Cyp26a1* continued to be highly expressed in the tailbud region of embryos without glucose supplementation (Fig 5A). Embryos cultured in serum supplemented with 2 mg/mL D-glucose exhibited minor caudal regression with diminished *Cyp26a1* expression in the tailbud region (Fig 5A). Embryos cultured in 3 mg/mL or 4 mg/mL D-glucose showed further reduction in *Cyp26a1* expression (Fig 5A), with the severity of caudal regression exhibiting a positive correlation with D-glucose concentrations.

Quantification *of Cyp26a1* expression showed a significant inverse correlation with the concentrations of supplemented D-glucose in both the tailbud region (Pearson coefficient *r* = -0.693 for 24 hrs and *r* = -0.931 for 48 hrs) (S2B Fig and Fig 5B) and the embryonic trunk (*r* = -0.911 for 24 hrs and *r* = -0.791 for 48 hrs) (S2C Fig and Fig 5C) with *p* < 0.001, which demonstrated that when the concentration of supplemented D-glucose increased, there was a corresponding linear decrease in *Cyp26a1* expression. However, no correlation was found between *Cyp26b1* and *Cyp26c1* expressions with D-glucose concentration, which further supported that the effect of D-glucose was specific to *Cyp26a1*.

## Discussion

In the present study, we have investigated whether elevated blood glucose is a critical factor by which maternal diabetes perturbs the gene expression of the RA catabolizing enzyme *Cyp26a1* in embryos, leading to an increased risk of birth defects. By lowering the blood glucose level in SD pregnant mice using PHZ, *Cyp26a1* expression in their embryos was up-regulated and restored to a level similar to embryos from ND mice (Fig 2). Such normalization of *Cyp26a1* expression could successfully increase the RA degrading activity in the embryos (Fig 3), resulting in a reduction of susceptibility to different types of RA-induced malformations (Fig 4). As a control, ND animals treated with PHZ did not exhibit any changes in maternal blood glucose level, nor any changes in *Cyp26a1* expression level and RA degrading activity in their embryos, which supported that glucose rather than PHZ is the factor regulating *Cyp26a1* expression. Furthermore, culturing rat embryos in serum containing varying concentrations of D-glucose led to a dose-dependent inhibition of the expression of *Cyp26a1* (S2 Fig, Fig 5). In both *in vivo* and *in vitro* experiments, expressions of *Cyp26b1* and *Cyp26c1* were unaffected. These results demonstrate that the expression of *Cyp26a1*, but not *Cyp26b1* or *Cyp26c1*, is inversely correlated with glucose concentration. Taken together, findings in this paper support that hyperglycemia is a critical factor that leads to increased susceptibility to various malformations via down-regulation of *Cyp26a1* and disruption of RA homeostasis.

Real-time quantitative RT-PCR results show that *Cyp26a1* is the only CYP26 family member predominantly expressed in the tailbud region of the embryo. Specific dysregulation of *Cyp26a1* in diabetic pregnancy will therefore render the caudal region highly sensitive to ectopic RA. However, in the anterior region of the mid-gestation embryo, *Cyp26a1* is co-expressed with *Cyp26c1* [23], while *Cyp26b1* is expressed in rhombomeres 3 and 5 [24]. Since both *Cyp26b1* and *Cyp26c1* may play a role in protecting tissues against the teratogenic effect of excess RA, in comparing the RA-degrading activity in the anterior region (Fig 3C), the difference between ND and SD groups could be dampened by these 2 subtypes. The basal expressions of two constitutively expressed genes, *Cyp26b1* and *Cyp26c1*, were not affected in the SD group, indicating that the altered expression of *Cyp26a1* was not due to global disruption of gene regulation in the embryo. CYP26s are the major RA catabolizing enzymes protecting specific tissues against overexposure to RA. The response and sensitivity of the three *Cyp26* subtypes to RA insult are different. *Cyp26a1* is highly sensitive to RA levels. Its expression in embryos was dramatically upregulated by 4-fold at 8 hrs after being challenged with 50 mg/kg RA on GD 9 (S3A Fig). *Cyp26a1* has two RA response elements in its promoter region, which act synergistically to provide a maximal response mediated via retinoic acid receptors RARγ/RARα [25, 26]. *Cyp26b1* is also sensitive to exogenous RA, and its expression was increased by 3-fold at 8 hrs after RA insult (S3B Fig). It is noted that the extent of up-regulation of *Cyp26a1* in embryos of SD mice is not as substantial as the ND group. This finding may explain why even a relatively small difference in the basal *Cyp26a1* expression between ND and SD group (Fig 2I and 2J) can lead to a marked difference in RA levels in the tissues upon challenged with RA (Fig 4B and 4C), leading to dramatic differences in susceptibility to an array of malformations (Fig 4D–4F). Interestingly, such differential up-regulation of *Cyp26a1* between ND and SD group in response to RA challenge is not apparent in *Cyp26b1* (S3B Fig). In contrast, *Cyp26c1* was significantly inhibited under RA insult (S3C Fig), which suggested that *Cyp26c1* may not be the critical enzyme that protects tissues in embryos against exogenous RA. Homozygous deletion of T-box 1 (*Tbx1*) in mouse embryos has been shown to suppress the expressions of *Cyp26a1*, *Cyp26b1* and *Cyp26c1* in pharyngeal tissues [27]. However, our data show that *Tbx1* expression in mouse embryos did not alter under maternal diabetes with or without PHZ treatment (S4 Fig), which are in line with the findings of unchanged *Cyp26b1* and *Cyp26c1* expressions in diabetic pregnancy. Moreover, *Tbx1* does not express in the tailbud region. Taken together, it is unlikely that down-regulation of *Cyp26a1* in embryos of diabetic pregnancy is mediated via dysregulation of *Tbx1*.

Excess RA is well-known to be teratogenic to embryos. However, a recent finding showed that administration of 3 mg/kg RA once daily to pregnant mice with gestational diabetes (GDM) could significantly reduce cardiac injury in pre- and post-delivery mother mice [28]. We have also previously demonstrated that administration of a low dose (0.625–1.25 mg/kg) of RA to pregnant mice with pregestational diabetes could upregulate *Cyp26a1* expression in their embryos. Such preconditioning with low dose RA could thereby create a protective effect against various types of malformations, such as caudal regression, exencephaly and spina bifida, induced by a teratogenic dose (25 mg/kg) of RA. Thus, whether RA has protective or deleterious effects will depend on its dosage. A low dose RA can restore the disrupted RA homeostasis in embryos of diabetic pregnancy and bring beneficial effects.

Glucose is an indispensable energy source during early embryogenesis. Any interruption in glucose utilization can initiate a cascade of events that leads to aberrant embryo development and congenital malformations [29]. In our studies, we examined embryos at an early stage of organogenesis because this is the stage that utilizes the highest amount of glucose [29] and is the most sensitive window for congenital malformations. Several primary glucose transporters (Slc2a1, Slc2a2, and Slc2a3) have been identified in early post-implantation embryos [30, 31]. When *Slc2a2*^+/−^ male and female mice were crossed and transient hyperglycemia was induced in pregnant mice with glucose injection, *Slc2a2*^+/−^ embryos were partially protected while *Slc2a2*^−*/*−^ embryos were completely protected from hyperglycemia-induced neural tube defects [32]. These findings support that Slc2a2 is the key glucose transporter for excessive glucose uptake in conditions of elevated glucose, which is one of the major causes of diabetic embryopathy.

RA distribution in the embryo is tightly controlled during embryogenesis by the combined action of synthesizing enzymes of the retinal dehydrogenase (RALDH) family and catabolic enzymes of the CYP26 family. Both types of enzymes are dynamically and spatially restricted during embryogenesis, and their expression domains are non-overlapping and complementary to each other [12]. It is well documented that hyperglycemia induces oxidative stress, and there is much evidence that implicates oxidative stress in the pathogenesis of diabetic embryopathy [33–37]. Inhibition of embryonic RA synthesis by aldehydes of lipid peroxidation has been demonstrated [38]. Aldehydes of lipid peroxidation, including trans-2-nonenal, nonyl aldehyde and 4-hydrozy-2-nonenal could inhibit all-*trans* RA synthesis from retinal. In contrast, addition of antioxidant reduced glutathione that conjugates with aldehydes could prevent the inhibition, suggesting that these aldehydes may compete with the RALDH to result in a mutual inhibition between oxidation of retinal and other aldehydes, leading to a deficiency of endogenous RA, particularly in the RALDH expressing tissues [38]. Combining this with our findings that RA is raised in CYP26-expressing tissues, it seems embryonic RA homeostasis is disrupted under diabetic conditions.

Undoubtedly, there are additional critical developmental control genes whose expressions are affected by maternal diabetes and lead to various types of malformations. RA is associated with spermatogonia differentiation [39], cancer risk [40], visual [41] and dermatologic disorders [42]. Coincidentally, diabetic patients are found to be associated with male infertility [43], increased cancer risk (e.g. pancreas, breast and prostate) [44–46], diabetic retinopathy and dermopathy. The involvement of retinoid homeostasis in these diabetic complications is worth to be investigated. In conclusion, our data support that maternal blood glucose levels in diabetic pregnancy regulate RA homeostasis. Instead of simply monitoring the glycemic level of the mother, it is important to monitor dietary retinoid intake and maternal retinoid circulation to reduce the risk of diabetic embryopathy.

## Supporting information

S1 Fig Illustration of (A) the tailbud region of a GD 9 embryo and (B) the tail length (TL) and crump-rump length (CRL) of a GD 18 fetus.
(TIF)

S2 Fig Whole embryo culture of GD 9 rat embryos in varying concentrations of D-glucose (D-Glu) for 24 hrs demonstrates a dose-dependent suppression of *Cyp26a1*. (A) Representative embryos showing the expression of *Cyp26a1* in the anterior and posterior regions of embryos detected by whole-mount in situ hybridization. Dose-dependent down-regulation of *Cyp26a1* in rat embryos cultured in varying concentrations (2, 3 and 4 mg/mL) of D-glucose (D-Glu) or an equivalent volume of DMEM as vehicle control (Control) for 24 hrs from GD 9 (equivalent to GD 7.5 of mouse embryo). Around 20–25 embryos from 3–4 litters in each group were examined. Scale bar representing 0.5 mm in embryos and 0.2 mm in tailbud region. Arrowhead indicated the optic primordium. (B-C) The mRNA expression levels of *Cyp26a1*, *Cyp26b1* and *Cyp26c1* relative to *β-actin* in the tailbud region of embryos (B) and embryonic trunk (C). Data are expressed as mean ± SEM with sample size = 4 for each group. Statistical analysis was conducted using Pearson’s correlation test.
(TIF)

S3 Fig Response and sensitivity of different Cyp26 subtypes to exogenous RA. (A-C) *Cyp26a1* (A), *Cyp26b1* (B) and *Cyp26c1* (C) gene expression in whole embryos of ND and SD mice with or without exposure to exogenous RA. Pregnant ND/SD mice received via intraperitoneal injection a single dose of 50 mg/kg bodyweight of RA (50RA) or an equivalent volume of vehicle as control (VEH) at GD 9 and gene expression was examined at 8 hrs after injection. Data are expressed as mean ± SEM with sample size = 3–4 for each group. Statistical analysis was conducted using two-way ANOVA followed by Bonferroni test.
(TIF)

S4 Fig *Tbx1* expression in GD 9 mouse embryonic trunk is neither altered under maternal diabetes nor PHZ treatment. (TIF)
