# Supplementary figures and images for: Hyperglycemia alters retinoic acid catabolism in embryos exposed to a maternal diabetic milieu
Source: PLoS One. 2023 Aug 24;18(8):e0287253. doi: 10.1371/journal.pone.0287253 (PMC10449132; doi:10.1371/journal.pone.0287253)

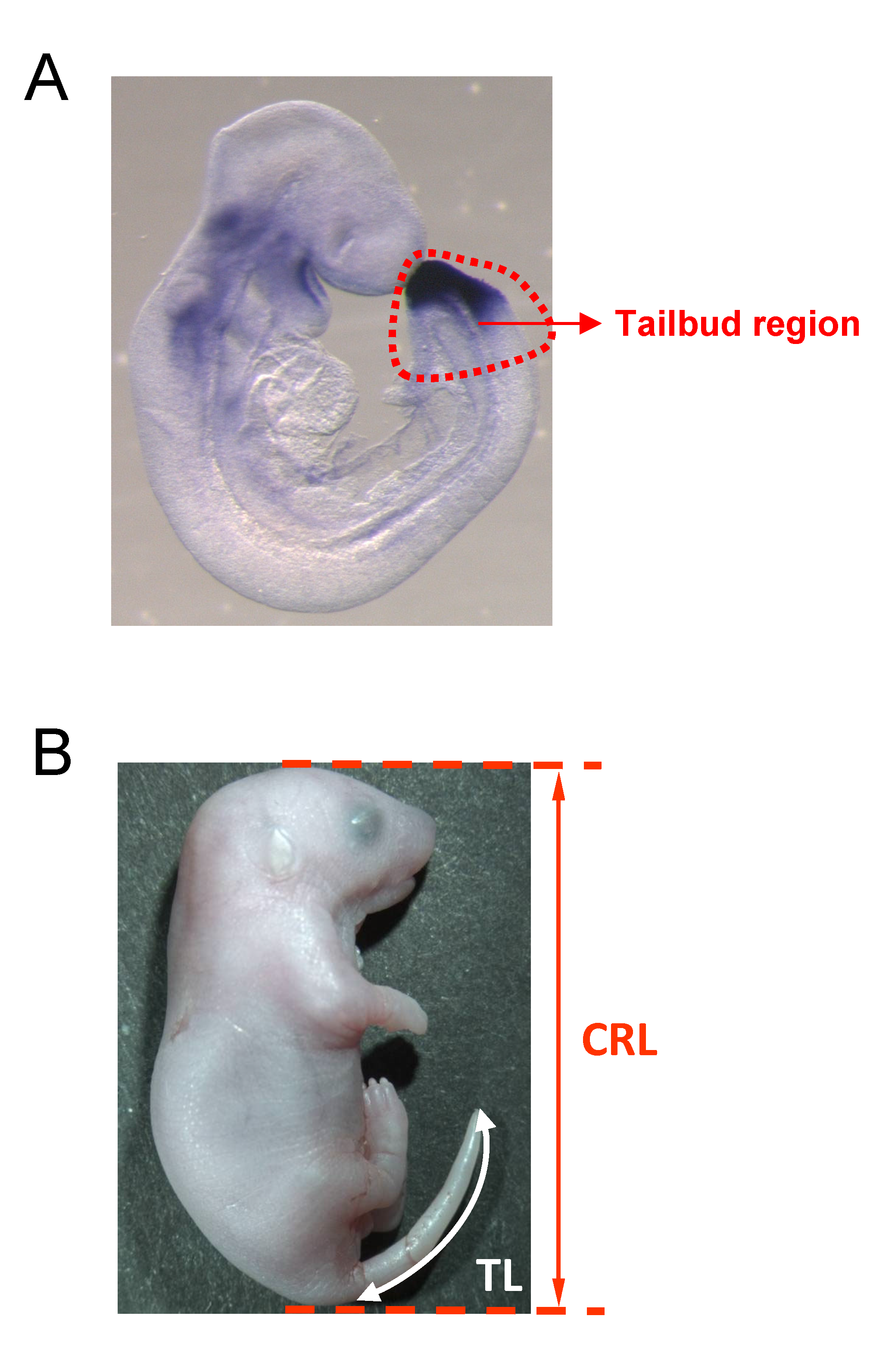

Supplement: S1 Fig — Illustration of (A) the tailbud region of a GD 9 embryo and (B) the tail length (TL) and crump-rump length (CRL) of a GD 18 fetus. (TIF) [file pone.0287253.s001.tif]

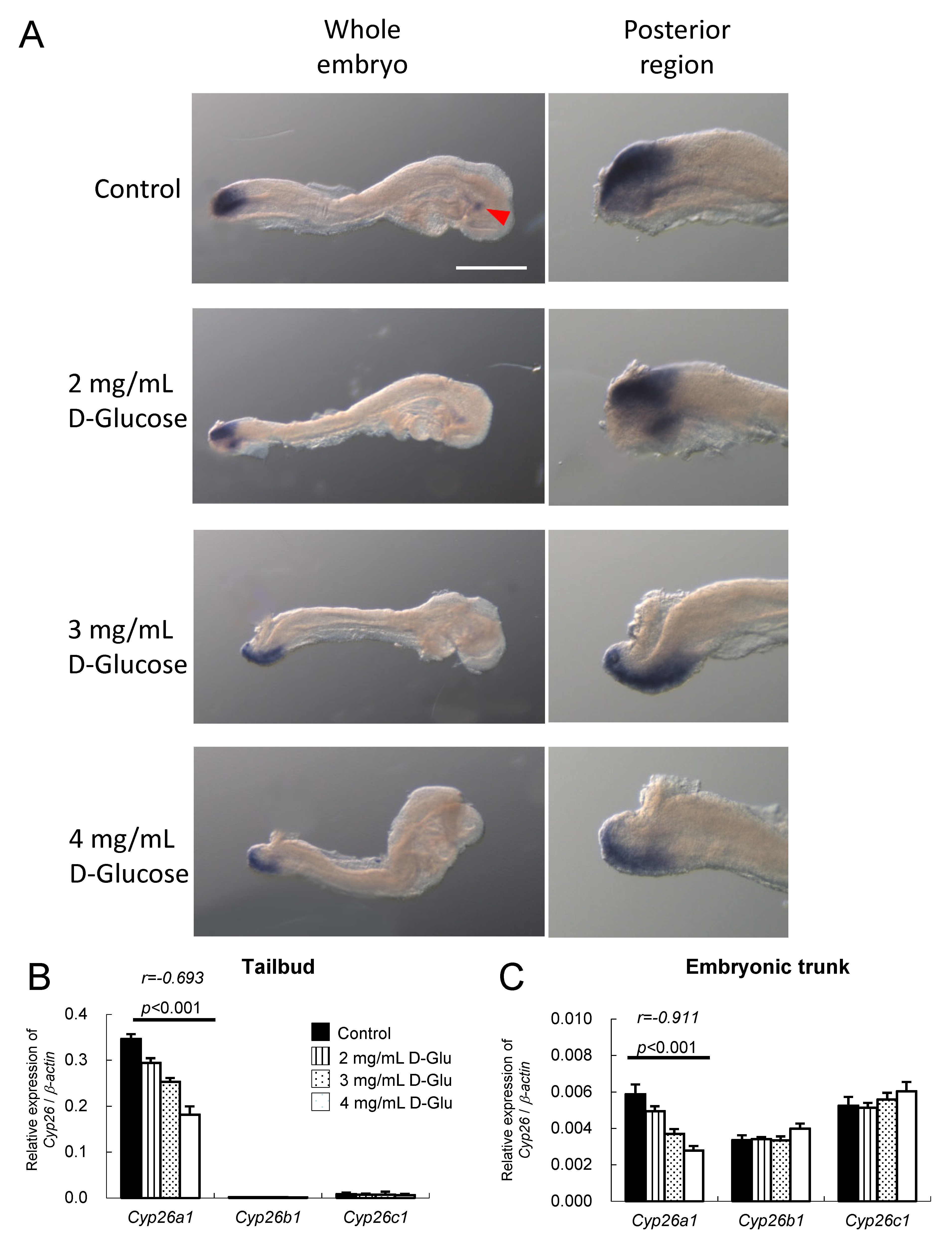

Supplement: S2 Fig — (A) Representative embryos showing the expression of Cyp26a1 in the anterior and posterior regions of embryos detected by whole-mount in situ hybridization. Dose-dependent down-regulation of Cyp26a1 in rat embryos cultured in varying concentrations (2, 3 and 4 mg/mL) of D-glucose (D-Glu) or an equivalent volume of DMEM as vehicle control (Control) for 24 hrs from GD 9 (equivalent to GD 7.5 of mouse embryo). Around 20–25 embryos from 3–4 litters in each group were examined. Scale bar representing 0.5 mm in embryos and 0.2 mm in tailbud region. Arrowhead indicated the optic primordium. (B-C) The mRNA expression levels of Cyp26a1, Cyp26b1 and Cyp26c1 relative to β-actin in the tailbud region of embryos (B) and embryonic trunk (C). Data are expressed as mean ± SEM with sample size = 4 for each group. Statistical analysis was conducted using Pearson’s correlation test. (TIF) [file pone.0287253.s002.tif]
